# The impact of telehealth remote patient monitoring on glycemic control in type 2 diabetes: a systematic review and meta-analysis of systematic reviews of randomised controlled trials

**DOI:** 10.1186/s12913-018-3274-8

**Published:** 2018-06-26

**Authors:** Puikwan A. Lee, Geva Greenfield, Yannis Pappas

**Affiliations:** 1 Department of Primary Care and Public Health, School of Public Health, Imperial College London, London, UK; 2 Institute for Health Research, University of Bedfordshire, Luton, UK

**Keywords:** Telehealth, Type 2 diabetes mellitus, Blood glucose, Remote

## Abstract

**Background:**

There is a growing body of evidence to support the use of telehealth in monitoring HbA1c levels in people living with type 2 diabetes. However, the overall magnitude of effect is yet unclear due to variable results reported in existing systematic reviews. The objective of this study is to conduct a systematic review and meta-analysis of systematic reviews of randomised controlled trials to create an evidence-base for the effectiveness of telehealth interventions on glycemic control in adults with type 2 diabetes.

**Methods:**

Electronic databases including The Cochrane Library, MEDLINE, EMBASE, HMIC, and PsychINFO were searched to identify relevant systematic reviews published between 1990 and April 2016, supplemented by references search from the relevant reviews. Two independent reviewers selected and reviewed the eligible studies. Of the 3279 references retrieved, 4 systematic reviews reporting in total 29 unique studies relevant to our review were included. Both conventional pairwise meta-analyses and network meta-analyses were performed.

**Results:**

Evidence from pooling four systematic reviews found that telehealth interventions produced a small but significant improvement in HbA1c levels compared with usual care (MD: -0.55, 95% CI: -0.73 to − 0.36). The greatest effect was seen in telephone-delivered interventions, followed by Internet blood glucose monitoring system interventions and lastly interventions involving automatic transmission of SMBG using a mobile phone or a telehealth unit.

**Conclusion:**

Current evidence suggests that telehealth is effective in controlling HbA1c levels in people living with type 2 diabetes. However there is need for better quality primary studies as well as systematic reviews of RCTs in order to confidently conclude on the impact of telehealth on glycemic control in type 2 diabetes.

**Electronic supplementary material:**

The online version of this article (10.1186/s12913-018-3274-8) contains supplementary material, which is available to authorized users.

## Background

Diabetes is a serious, chronic condition that is recognised as an important cause of premature death and disability worldwide. In particular, the prevalence of type 2 diabetes is emerging as one of the greatest global public health challenges in twenty-first century [1]. In the UK, the National Health Service (NHS) spends around £9.8 billion a year on diabetes. Most of this cost (80%) is spent on treating complications alone as a result of poorly controlled diabetes, of which many are possibly preventable [2]. These could include blindness, kidney failure, heart attacks, strokes and amputations [2]. Diabetes UK warned that “diabetes is threatening to bankrupt the NHS after a 60% rise in cases in the past 10 years”. The cost of treating diabetes complications is also expected to almost double by 2035/6 if no actions are taken to prevent these complications [3]. The urgent need for improvements in effective management of diabetes and preventing its complications is therefore evident.

The aim of diabetes management is to keep blood sugar levels as close to normal as possible to improve symptoms and minimise the risk of long-term complications [4]. This requires close monitoring of vital signs and effective working relationship between the patient and their healthcare professionals. The provision of conventional outpatient care alone, which generally occurs less than 3 times a year [5], is therefore not sufficient.

There is a growing body of evidence that supports the uses of advanced and innovative technologies, such as telehealth, to monitor and manage people with diabetes at a distance and as frequently as it is needed [5–7]. Telehealth is generally described as the exchange of medical information from one location to another using electronic communications or digital technologies, such as desktop, laptop, mobile phones and other wireless tools [8].

Overall, existing evidence suggests that telehealth has the potential in improving HbA1c for patients living with diabetes but the overall magnitude of effect is unclear due to variable results reported in existing systematic reviews. Given that the literature already contains multiple systematic reviews on telehealth and type 2 diabetes [7, 9, 10], there is an opportunity to pool the evidence from all existing reviews to report an estimate of effect. Therefore, to create an evidence base for the effectiveness of telehealth on glycemic control in type 2 diabetes, we conducted the first systematic review of systematic reviews and meta-analyses of randomised controlled trials (RCTs) to assess the evidence of the effects of telehealth interventions on glycemic control in patients living with type 2 diabetes.

For the purpose of this study, we defined telehealth as remote patient monitoring (RPM), which involves the transmission (electronic or verbal) of self-monitored blood glucose (SMBG) readings to a healthcare professional or a specialist team at an offsite monitoring center for evaluation and feedback.

## Methods

### Inclusion criteria

Studies that met the following criteria were included in this review: i) systematic reviews and/or meta-analyses of RCTs with our definition of telehealth as an intervention; ii) adults ≥18 years of age with a diagnosis of type 2 diabetes; iii) comparison of standard outpatient care (usual care) or other RPM telehealth interventions; and iv) reported HbA1c outcome. Systematic reviews and/or meta-analyses of RCTs on RPM telehealth interventions were excluded if they: i) were non-English publications; ii) included a mixed study population (type 1 and type 2 diabetes) and results were not reported separately for type 2 diabetes; or iii) do not provide feedback to patients following the transmission of SMBG data. In systematic reviews where RPM telehealth was one part of a wider intervention, these were only included where the effects of the RPM telehealth component were individually reported. In addition, if the same authors had produced several publications of the same review, the most updated and/or the full report of the review were included, and other versions excluded.

### Search strategy

The literature search was conducted from April 1 to 8, 2016 and the electronic bibliographic databases including The Cochrane Library, MEDLINE, EMBASE, HMIC and PsychINFO were searched. All searches were restricted by date range to 1990 – April 2016. Limiting the search period from 1990 is likely to identify all apart from a very small minority of systematic reviews that were carried out earlier [11, 12]. A base strategy (see Additional file 1) was developed in MEDLINE (Ovid interface). This strategy was then converted to run effectively in other databases using different interfaces. Reference lists of all potentially relevant systematic reviews identified by the electronic searches were also checked for any eligible reviews that have not been identified in the search.

### Study selection and data extraction

Based on the eligibility criteria, two reviewers (AL and YP) independently screened the list of titles/abstracts identified through searches for systematic reviews. Selected systematic reviews at this stage were further included for a full-text review by the same two reviewers. Any disagreements between the reviewers about the inclusion and exclusion were resolved by discussion until a consensus was reached. The same two reviewers using Eppi-Reviewer software 4 then extracted data from the resulting final list of selected systematic reviews independently. The two sets of extracted data were then compared for quality and validity purposes. Consensus was achieved without negotiation.

### Assessment of risk of bias

The assessment of the methodological quality and strength of each systematic review was based on the AMSTAR tool, which is a validated measurement tool available for evaluating multiple systematic reviews [13]. The AMSTAR tool is a questionnaire that comprises of 11 criteria, which specifically assess the presence of: i) an a priori design; ii) duplicate study selection and data extraction; iii) a comprehensive literature search; iv) the use of status of publication as an inclusion criteria; v) a list of included/excluded studies; vi) characteristics of included studies; vii) documented assessment of the scientific quality of included studies; viii) appropriate use of the scientific quality in forming conclusions; ix) the appropriate use of methods to combine findings of studies; x) assessment of the likelihood of publication bias; and xi) documentation of conflict of interest [14].

Each of the 11 items is given a score of 1 if the specific criterion is met by a “yes” answer, or a score of 0 if the criterion is not met, unclear or not applicable. The overall AMSTAR score is calculated by adding all the individual item scores together. As defined by AMSTAR, quality is categorised into three levels: high quality if the total score is between 8 and 11, medium quality if the total score is between 4 and 7, and low quality if the total score is between 0 and 3.

The same two reviewers independently assessed each potentially relevant review for inclusion. Any disagreements between the reviewers were resolved by discussion and when required, a final opinion from a third reviewer was sought.

### Data analysis

To examine the overall magnitude of effect in using telehealth for controlling HbA1c levels in Type 2 diabetes, where possible, we conducted conventional pairwise meta-analyses as well as network meta-analyses (NMAs) of the included reviews. While the pairwise meta-analyses allowed us to investigate the difference of effect between telehealth interventions vs. usual care, the NMAs enabled us to explore if there is any specific telehealth application that is superior. NMAs involve the statistical combination of both direct and indirect evidence about pairs of interventions that originate from two or more separate studies to provide estimates of relative effectiveness for all comparators.

Care was taken to not include data from individual studies more than once by unpicking each of the included reviews and the subsequent combination of data of the individual primary studies included in the reviews. For HbA1c, where change from baseline data were reported in the trials and were accompanied by a measure of variation (for example standard deviation), these were extracted and used in the meta-analyses. Where measures of spread for change from baseline values were not reported, these trials were excluded from the meta-analyses.

Furthermore, due to the various telehealth applications (technologies) used as well as feedback methods provided in the interventions, we performed subgroup meta-analyses to assess whether their impact on glycemic control differed.

The conventional pairwise meta-analyses were conducted with reference to the Cochrane Handbook for Systematic Reviews, whereas NMAs were undertaken using the Netmeta package in R3.2.2. This uses a graph-theoretical method, which is mathematically equivalent to the frequentist network meta-analysis [15]. Heterogeneity was assessed using the overall *I*^2^ value for the whole network, which is a weighted average of the *I*^2^ value for all comparisons where there are multiple trials (both direct and indirect), and random-effects models were used if the *I*^2^ value was above 50% (as for pairwise meta-analyses, this was interpreted as showing the assumption of a shared underlying mean was not met, and therefore a fixed-effects model was inappropriate). A funnel plot generated in Review Manager 5 (RevMan 5) for HbA1c was used to visually assess publication bias.

Items on the PRISMA (Preferred Reporting Items for Systematic Reviews and Meta-Analyses) checklist that is relevant for a systematic review of reviews was used to report the findings (see Additional file 2).

## Results

### Search results

**Fig. 1:**
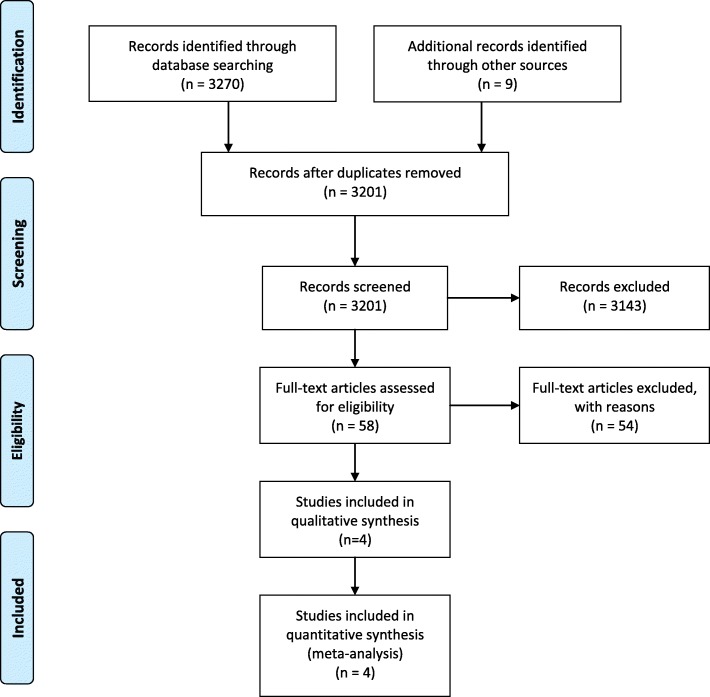
PRISMA flow diagram

The systematic literature search identified in total 3279 potentially relevant studies. After removing duplicate studies, 3201 studies were screened on their titles and abstracts for relevance. In total, 3143 were excluded because they were not systematic reviews or meta-analyses, or did not include telehealth interventions or a population with diabetes. For the remaining 58 studies, full text articles were obtained and reviewed against the inclusion and exclusion criteria. Overall, 54 studies did not meet the eligibility criteria such as being a systematic review and/or meta-analysis of RCTs, including telehealth interventions that met our definition of telehealth, and/or reporting Type 2 diabetes results separately. These studies were therefore not included in this review. A detailed list of excluded studies and reasons for their exclusion is provided in an additional file (see Additional file 3). Figure 1 provides the systematic review of reviews study flow chart that demonstrates the inclusion and exclusion process and results.

In total, four systematic reviews and/or meta-analyses met the inclusion criteria and were included in this review. Summaries of these are presented in evidence tables (see Additional file 4). The included reviews were published between the years 2009 and 2015. Only one review conducted meta-analyses [7].

The reviews we included and coded only assessed studies with an RCT design. Two of the four reviews included both type 1 and type 2 diabetes but only data for type 2 diabetes was used in this review.

Although all four reviews focused on determining the effectiveness of telehealth applications for individuals with diabetes, the scopes of the reviews varied. One review solely targeted telehealth remote patient monitoring interventions that incorporated key elements of structured self-monitoring of blood glucose [16]. Another review only focused on telehealth interventions in patients with type 2 diabetes and inadequate glycemic control [7]. For the remaining two reviews, one included studies using cell phones and wireless devices only [17], and the other one looked at studies on Internet blood glucose monitoring systems only [18]. Furthermore, one of the four reviews only included patients with type 2 diabetes using insulin [18], two reviews included both insulin- and non-insulin-dependent patients with type 2 diabetes [16, 17] and one review provided no details on this [7]. The two reviews that included a mixed population of insulin- and non-insulin-dependent patients did not report any results separately for the two groups.

In total, we found 78 studies coded in the four reviews, of which 51 were considered relevant to our review. Individual telehealth interventions that were deemed irrelevant and excluded included studies that did not involve or report results separately for participants with type 2 diabetes, studies that did not include telehealth interventions that met our review’s definition of telehealth or studies that did not involve the transmission of SMBG data followed by automatic and/or healthcare provider feedback. The 51 relevant studies contained 16 duplicates and four studies also had multiple publications (*n* = 10); thus, we identified in total 29 unique studies relevant to our review. However, four of these studies did not provide extractable data for HbA1c and were therefore not included in our meta-analyses. The number of relevant trials included in each review ranged from five to 18 trials and the sample sizes of the various trials ranged widely from 30 to 1665 participants. The length of the interventions ranged from 3 to 60 months, with majority of the interventions lasting three months (13/29 studies) or between six and 12 months (13/29 studies). Only one study reported a five-year follow-up.

Moreover, studies also varied in intervention complexity; nearly half of the studies involved automatic transmission, where self-monitored data are transmitted directly and automatically to a receiving station without interruption. This typically involves patients using either a mobile phone with a diabetes management software installed and connected to a blood glucose meter or a telehealth unit that is connected by a secure computer network at home. Approximately one third of studies used the Internet or a website to deliver self-monitored blood glucose results and self-management information. Lastly, there were also some interventions delivered by telephone. Telephone-delivered interventions do not require patients to electronically transmit their daily blood glucose readings to their healthcare professionals. Instead, they typically require patients to log their blood glucose levels daily and a healthcare professional would follow up with a telephone call weekly to review the blood glucose log and discuss the glucose values with the patients.

Moreover, when it came to providing feedback to patients; majority of the interventions provided feedback at least once daily, if not more, using one or a combination of feedback methods including, SMS or text messaging to the patient’s mobile phone, messaging through internet, telephone calls and/or secure messages via a patient portal or through a telehealth system. Majority of the feedback was provided by a healthcare professional but nearly half of the studies provided automatic feedback generated from computer algorithms, without provider input. Only one study utilised videoconferencing as a way of delivering feedback to patients and three studies only contacted patients with feedback if necessary (i.e. when blood glucose levels were not within normal range). In addition to the wide range of technologies used, many of the studies also incorporated an educational component to their telehealth intervention to improve patients’ knowledge in diabetes self-management. Almost all studies indicated that the transmitted self-monitored blood glucose data were used to provide feedback, or modify treatment or behavior, although the details varied.

For the purposes of subgroup meta-analyses, telehealth applications and feedback methods were classified into different categories. For telehealth applications, these were grouped into four categories according to the method of transmission used for transmitting self-monitored data to a receiving station remotely: (i) Internet/web (including any application or software on a computer or a mobile phone that uses data networks or the Internet); (ii) automatic transmission (including the use of any telehealth unit placed at home that automatically and directly transmit data upon taking measurements); (iii) automatic mobile transmission (including the use of any telehealth equipment that allows for the direct transmission of self-monitored data on the move, without interruption); and (iv) telephone (interventions delivered by regular telephone calls from a healthcare professional, no electronic transmission of data involved).

With regards to feedback methods, six categories were classified: (i) automated message (automated messaging generated from computer algorithms, without healthcare provider input); (ii) human calls (interactive telephone calls with a healthcare provider or researcher); (iii) human calls only if necessary (i.e. interactive telephone calls with a healthcare provider only when blood glucose levels were outside of normal range); (iv) human message (personalised feedback via messaging from a healthcare provider); (v) human message + calls (personalised feedback via messaging from a healthcare provider followed up by an interactive telephone call); and (vi) videoconferencing (use of video telecommunication technologies which allow the patient to communicate in real-time with a healthcare provider at a distance). Automated and human messages could include messaging through Internet, SMS, a patient portal and/or a telehealth system.

### Quality assessment of reviews and meta-analyses

Among the four systematic reviews described in the current review, two were rated moderate quality reviews [7, 17]. The methodological quality of the remaining two reviews [16, 18] was considered low according to the AMSTAR tool (total score of 1 and 2, respectively). The most common methodological weaknesses were lack of including an ‘a priori’ design, a list of both included and excluded studies, and a search for “grey literature or unpublished literature” and/or detail the source of funding/support for the systematic review and for each of the included studies. For the two reviews that scored a low rating, in addition to the above, the authors did not provide details on whether they performed duplicate study selection and data extraction procedures nor include the use of any quality scoring tool or checklist. Furthermore, three of the four included reviews were also of qualitative nature; hence further 2 out of the maximum 11 points were lost due to the lack of any statistical pooling of results and statistical assessment for the presence of publication bias. The reason for not conducting meta-analyses in the three qualitative reviews was not described.

In the two reviews [7, 17] that used a quality scoring tool (Downs and Black score and Jadad score, respectively) to assess the scientific quality of their included RCTs, 18 out of 25 studies were rated as moderate/good quality. The remaining five RCTs were rated as low quality based on the Jadad score.

### Effectiveness of telehealth interventions in type 2 diabetes

All four reviews primarily examined the effect of telehealth on HbA1c. Russel-Minda et al., 2008 reported that three out of their five studies on type 2 diabetes using cell phones with SMS and Internet (some with nurse-directed educational component) found a statistically significant improvement in HbA1c when compared to usual care. Tildesley et al., 2015 who identified nine randomised controlled Internet blood glucose monitoring systems (IBGMS) trials, reported that eight of them showed significantly improved HbA1c levels in the IBGMS group when compared with the usual care group. However, one of the studies only achieved significant HbA1c reduction at six months but not 12 months. Greenwood and colleagues (2014) identified and reviewed 16 teleheatlh remote patient monitoring interventions using one or a combination of technologies (including telephone, mobile phone, wireless device, telehealth unit and/or internet), that incorporated key elements of structured self-monitoring of blood glucose (SMBG) identified as essential for improving HbA1c. They reported that, compared to usual care, telehealth was shown to significantly improve HbA1c in seven out of the 15 reviewed studies. The authors also found that interventions that incorporated at least five out of the seven key elements of structured SMBG consistently achieved significant HbA1c improvements between study groups. In addition, studies that incorporated at least four of the seven key elements of structured SMBG and had a baseline HbA1c greater than 8% resulted in a decrease of at least 0.7% in HbA1c levels. Lastly, Huang et al., 2015 reported that, compared to usual care, 11 out of the 18 studies included in the review found a statistically significant improvement in HbA1c in the telehealth group. Furthermore, a meta-analysis of the 18 studies found that participants using telehealth had significantly improved HbA1c levels when compared to participants receiving usual care (MD = − 0.54, 95% CI: -0.75 to − 0.34). The same review also conducted subgroup analyses that included feedback methods, duration of follow-up, study location, baseline HbA1c and sample size. They found that feedback by interactive telephone calls with a healthcare provider or researcher to be associated with the greatest improvement in HbA1c (K 1.13; 95% CI, K 1.51 to K 0.75), followed by automated phone-based SMS and/or internet-based messaging (K 0.36; 95% CI K 0.47 to O 0.24). No improvement in HbA1c was reported with automated telephone calls (K 0.01; 95% CI K 0.32 to K 0.29). For the remaining subgroup analyses, a significant reduction in HbA1c was reported to be associated with Asian ethnic groups, small study sample sizes, and patients with a baseline HbA1c level of 8% or higher.

In order to determine the overall effectiveness of telehealth on glycemic control in individuals with type 2 diabetes, we conducted additional meta-analyses that incorporated all the unique studies, with extractable data on HbA1c, identified in the four reviews.

**Fig. 2:**
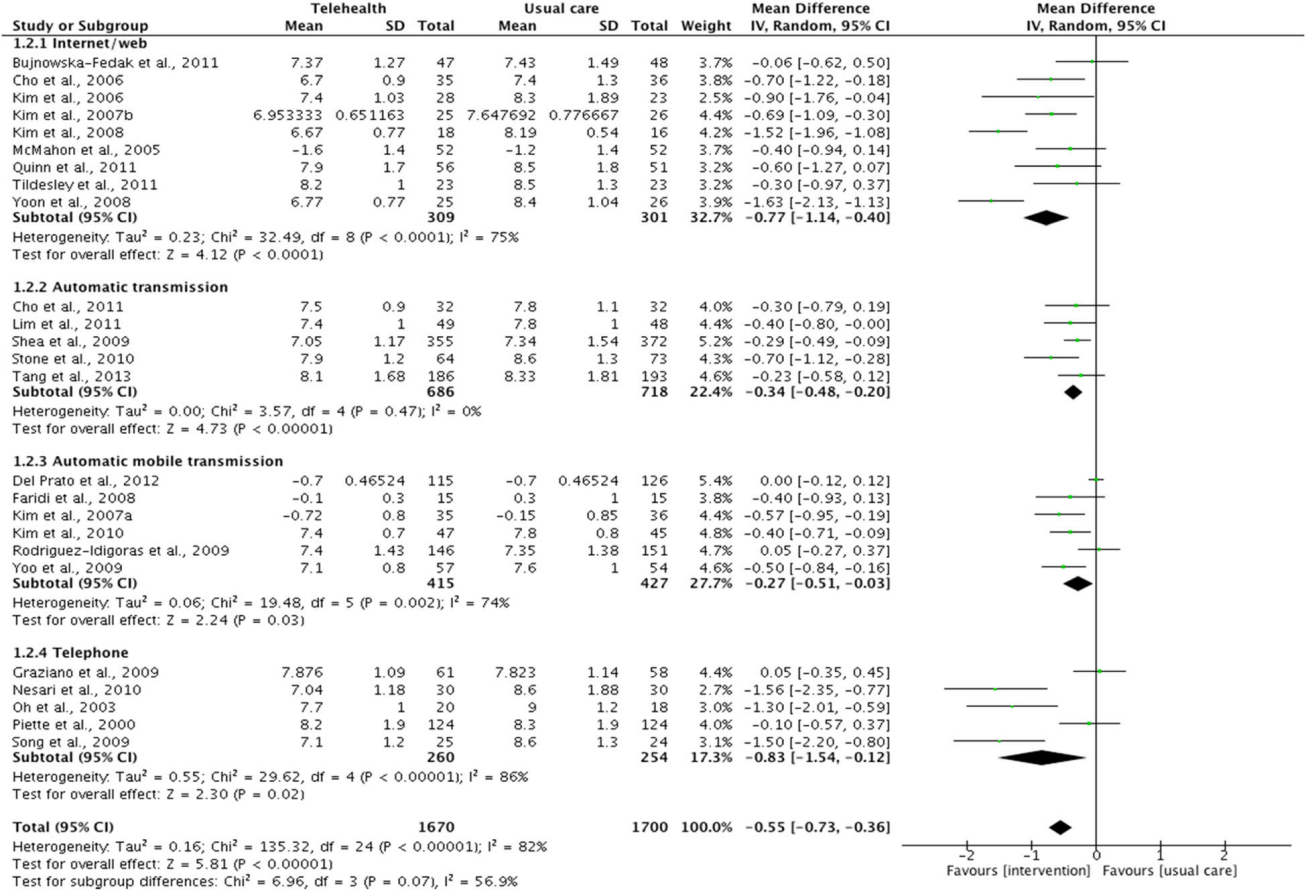
Pairwise meta-analyses on HbA1c by telehealth applications/ transmission methods

A pairwise meta-analysis pooling evidence from 25 (out of 29) RCTs indicate that, compared to usual care, telehealth is associated with significant improvements in HbA1c in patients with type 2 diabetes (MD = − 0.55, 95% CI: -0.73 to − 0.36) but with statistical heterogeneity to the variability in effect estimate (I^2^ = 82%; Fig. 2). In addition, although telehealth was statistically better than usual care in improving HbA1c levels, the confidence interval of the mean difference crossed the threshold for minimal clinically important difference (MID) as defined by the NICE guidelines on type 2 diabetes in adults [19]. The greatest effect was seen in telephone-delivered interventions (MD = − 0.83, 95% CI: -1.54 to − 0.12), followed by Internet blood glucose monitoring system interventions (Internet/web) (MD = − 0.77, 95% CI: -1.14 to − 0.40). The effect of automatic data transmission using a mobile phone or a telehealth unit was shown to be similar (MD = − 0.27, 95% CI: -0.51 to − 0.03 vs. MD = − 0.34, 95% CI: -0.48 to − 0.20). Moreover, significant heterogeneity was reported in all subgroups except from the ‘automatic transmission’ subgroup (Fig. 2).

A network meta-analysis of the 25 RCTs further indicated that all telehealth interventions provide a significant lowering of HbA1c compared with usual care, with Internet blood glucose monitoring system interventions also providing significantly more lowering of HbA1c than telehealth interventions using automatic mobile transmission (MD = − 0.4934, 95% CI: -0.9250 to − 0.0619). However, considerable between-study heterogeneity was present (I^2^ = 75.3%; see Additional file 5).

**Fig. 3:**
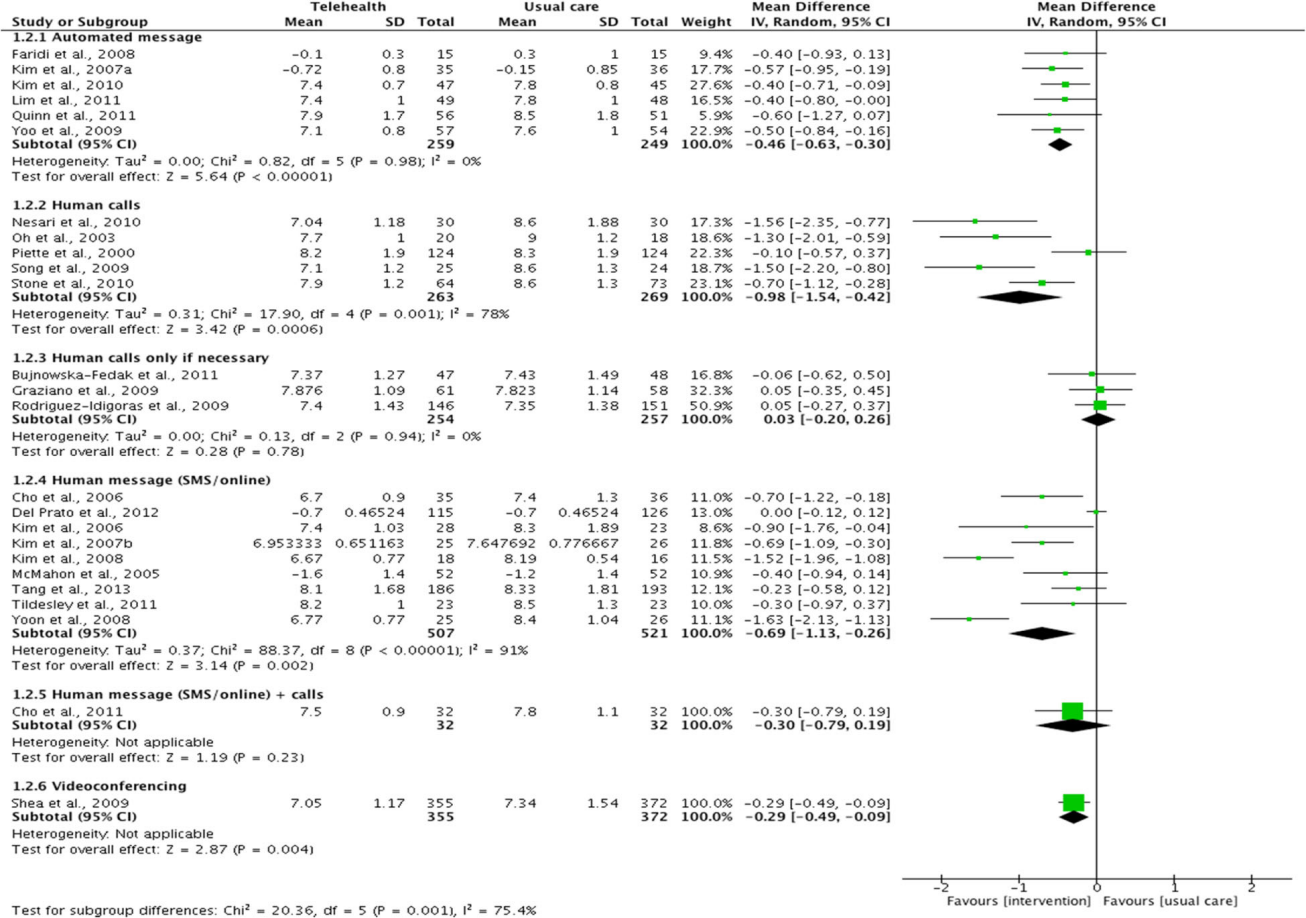
Pairwise meta-analyses on HbA1c by telehealth feedback methods

We also conducted pairwise and network meta-analyses on feedback methods. Evidence from pairwise meta-analyses of 25 RCTs showed that the human calls subgroup was associated with the greatest effect size (MD: -0.98; 95% CI: -1.54 to − 0.42), followed by human message (MD: -0.69; 95% CI: -1.13 to − 0.26) and then automated message (MD: -0.46; 95% CI: -0.63 to − 0.30) (Fig. 3). Very small effect sizes or no improvements were reported for feedback via human message + calls (*n* = 1), videoconferencing (*n* = 1) and human calls only if necessary (*n* = 3). This is most likely due to the very limited number of studies being available in these subgroups. Similar results were reported in the NMA (see Additional file 6), where significant reduction in HbA1c levels was associated human calls, human message and automated message subgroups when compared with usual care groups. In addition, the NMA also suggested that feedback provided through human calls and human message to significantly improve hbA1c levels compared with feedback provided by healthcare providers only when HbA1c levels fall outside of normal range (MD: -0.9768, 95% CI: -1.7278 to − 0.2285 and MD: -0.7031, 95% CI: -1.3697 to − 0.0365, respectively).

### Risk of bias

A funnel plot generated in Review Manager assessed publication bias and significant publication bias towards positive outcomes in the included studies was observed.

## Discussion

Telehealth, which can be defined as personalised healthcare delivered at a distance, is believed to have the potential to enhance the quality of healthcare. Over the last decade, there have been numerous studies aimed at assessing the feasibility and effectiveness of telehealth strategies on the management of diabetes [17]. As the number of published telehealth studies began to increase, a plethora of systematic reviews on telehealth interventions of variable scope and quality, also began to emerge.

Hence, in order to create an evidence-base for the effectiveness of telehealth on glycemic control in type 2 diabetes specifically, we conducted a review of systematic reviews. Moreover, in order to generate precise and reliable conclusions; we specifically focused on telehealth applications that involved patients transmitting (electronically or verbally) SMBG results to a receiving station or person to receive automated messages and/or healthcare provider feedback.

Our systematic literature search identified, in total, 58 potential telehealth and diabetes systematic reviews but only four reviews met our inclusion criteria, of which two were of moderate quality and the other two of low quality according to the AMSTAR tool. All four reviews concluded that telehealth interventions have the potential in improving glycemic control in people with type 2 diabetes. However, when we pooled the HbA1c results from the 25 RCTs included in the four reviews together, only 14 (56%) studies reported a significant improvement in telehealth intervention versus usual care group.

The greatest improvements in glycemic control with telehealth was reported in studies where participants had a mean baseline HbA1c level of 8% or greater, regardless if they were on insulin or not [7, 16, 18]. These findings were similar to other recent published systematic reviews related to telehealth and diabetes management [20–22]. Where study participants had a mean baseline HbA1c at or near their glycemic target, small but significant improvements were also reported [18], suggesting that glycemic improvements with telehealth is not limited to patients with type 2 diabetes and inadequate symptomatic control at baseline (≥8%) only.

It is important to note when interpreting the results from our review that this review is limited to capturing and reporting information presented in the included systematic reviews and meta-analyses. Two important limitations therefore exist in our review of systematic reviews. Firstly, we depended on the authors of the four included reviews in this review to have adequately included and critically appraised individual studies as well as correctly captured and interpreted the study results. We did not examine the full-texts of individual studies unless there were major data gaps we had to fill in the reviews or we felt that there may be discrepancies in the analyses of individual studies included in multiple reviews. Hence, potential omissions or errors that may be present in our coding and/or analyses and results, may be due to unreported errors in the original reviews and/or original primary studies included in those reviews.

Secondly, the four reviews varied greatly in terms of the type of telehealth interventions, duration of follow-up, study sample size, baseline HbA1c levels, and/or insulin- and non-insulin-dependent population with type 2 diabetes. In addition, the reviews included minimal description on the additional telehealth components such as e-learning, virtual coach and/or networking support group and how these additional components may have impacted on the health outcomes. Details on feedback frequency and how it was used to help support and improve patient self-management skills were also limited. In addition, only one of the four reviews conducted meta-analyses. Hence, statistical pooling of results to assess the estimated mean effect of HbA1c with telehealth is limited. Although we attempted to pool and examine the findings on HbA1c from the four reviews, substantial heterogeneity among individual studies was evident from the overall meta-analyses, majority of the subgroup analyses (by transmission and feedback methods) as well as network meta-analyses due to the diversity of telehealth interventions and applications used in the trials. It is therefore difficult to confidently conclude which telehealth component or type 2 diabetes population is likely to benefit the most and from which telehealth intervention, especially in the long-term.

We therefore agree with the recommendations made by the authors of the four reviews that more high quality, well-designed RCTs with large sample sizes and longer follow-up durations are needed to investigate the sustainability and to confirm the benefits of telehealth in type 2 diabetes management. In addition, to produce reliable pooled estimates of HbA1c, it would be useful for future studies to take into account the differences in baseline HbA1c level when recruiting study participants and drawing conclusions from findings. Greenwood et al., (2014) have also suggested that future telehealth research should explore the use and impact of telehealth on behavior change in people with non-insulin-dependent type 2 diabetes. This group of users would primarily use telehealth for lifestyle and behavior change to manage their diabetes in contrast to insulin users who would primarily use telehealth for monitoring and adjusting insulin treatment. Comparing the usage and impact of telehealth in these two sub populations of diabetes may provide some explanations as to which and how different telehealth components work and/or are responsible for improved glycemic control for people with diabetes. For example, are improvements in HbA1c level with telehealth dependent on insulin dose adjustments, or SMBG frequency or from increased self-motivation and/or patient-physician communication? Future research in this area could provide important knowledge for clinical practice for diabetes management [16].

To further strengthen the current evidence-base for telehealth and the management of type 2 diabetes, future reviews should also consider assessing the cost-effectiveness and outcome measures that may influence the uptake and outcomes of telehealth interventions, such as healthcare provider satisfaction and patient health-related outcomes (e.g. quality of life and quality of care) as well as the usability and feasibility of self-monitored devices for diabetes management. These are all important evidence for current clinical guidelines and health-related economic policies.

## Conclusion

This review found that telehealth interventions produced a small but significant improvement in HbA1c levels compared with usual care, suggesting that telehealth has the potential to deliver beneficial change. However, there is a need for higher quality primary studies as well as systematic reviews of RCTs in order for us to draw any definite conclusions. Furthermore, in order to provide a complete evidence base for policy makers on the overall effectiveness of telehealth interventions for type 2 diabetes management, future reviews should also focus on the impact of telehealth in other areas of diabetes management such as quality of life, quality of care and cost-effectiveness.

## Additional files

Additional file 1: Base literature search strategy. (DOC 37 kb)

Additional file 2: PRISMA checklist. (DOC 62 kb)

Additional file 3: Excluded references. (DOCX 119 kb)

Additional file 4: Evidence tables. (DOCX 98 kb)

Additional file 5: NMAs on HbA1c by telehealth transmission methods. (DOCX 89 kb)

Additional file 6: NMAs on HbA1c by telehealth feedback methods. (DOCX 119 kb)

## Abbreviations

IBGMS: Internet blood glucose monitoring systems
MID: Minimal clinically important difference
NHS: National health service
PRISMA: Preferred Reporting Items for Systematic Reviews and Meta-Analyses
RCT: Randomised controlled trial
RPM: Remote patient monitoring
SMBG: Self-monitored blood glucose
