# Supplementary material for: The impact of telehealth remote patient monitoring on glycemic control in type 2 diabetes: a systematic review and meta-analysis of systematic reviews of randomised controlled trials
Source: BMC Health Serv Res. 2018 Jun 26;18:495. doi: 10.1186/s12913-018-3274-8 (PMC6019730; doi:10.1186/s12913-018-3274-8)
Supplement: Supplementary file 1 — Base literature search strategy. (DOC 37 kb) [file 12913_2018_3274_MOESM1_ESM.doc]

**Additional file 1. Base literature search strategy**

| 1. exp Diabetes Mellitus/ |
| --- |
| 1. (diabetes or diabetic* or NIDDM or IDDM or MODY).ti,ab. |
| 1. 1 or 2 |
| 1. exp Telecommunications/ |
| 1. exp Computer Communication Networks/ |
| 1. (telematic or tele-matic or telemanagement or tele-management or telenursing or tele-nursing or telerehab* or tele-rehab* or teleservic* or tele-servic* or telemedic* or tele-medic* or telehealth* or tele-health or telecare or tele-care or tele-home or telehome* or telemonitor* or tele-monitor* or telecommunication* or tele- communication* or teleconferenc* or tele-conferenc* or tele-consult* or teleconsult* or email or e-mail or electronic mail or online or web or web-based or internet or internet-based or e-health or ehealth or telephone or videoconferenc* or video-conferenc*).mp. |
| 1. ((remote or wireless or mobile or cellular or telephone) adj2 (monitor* or consult* or manag*)).mp. |
| 1. or/4-7 |
| 1. 3 and 8 |
| 1. (meta analy$ or metaanaly$ or pooled analysis or (systematic$ adj2 review$)).mp. or (published studies or published literature or medline or embase or cochrane).ab. |
| 1. (review or overview or (evidence adj base$) or evidence-base*).ti,ab. |
| 1. 10 or 11 |
| 1. 9 and 12 |
| 1. limit 13 to (english language and humans and yr="1990 - 2013") |
