# Supplementary material for: The impact of telehealth remote patient monitoring on glycemic control in type 2 diabetes: a systematic review and meta-analysis of systematic reviews of randomised controlled trials
Source: BMC Health Serv Res. 2018 Jun 26;18:495. doi: 10.1186/s12913-018-3274-8 (PMC6019730; doi:10.1186/s12913-018-3274-8)
Supplement: Supplementary file 3 — Excluded references. (DOCX 119 kb) [file 12913_2018_3274_MOESM3_ESM.docx]

**Additional file 3. Excluded references from the review of systematic review**

| **Reference** | **Reason for exclusion** |
| --- | --- |
| 1. Aalbers T, Baars MAE, Rikkert MGMO. Characteristics of effective Internet-mediated interventions to change lifestyle in people aged 50 and older: A systematic review. Ageing Res Rev [Internet]. 2011;10(4):487–97. Available from: http://dx.doi.org/10.1016/j.arr.2011.05.001 | No relevant outcomes and T2DM outcomes not reported separately |
| 2. Adaji A, Schattner P, Jones K. The use of information technology to enhance diabetes management in primary care: a literature review. Inform Prim Care [Internet]. 2008;16(3):229–37. Available from: http://hijournal.bcs.org/index.php/jhi/article/view/698%5Cnhttp://search.ebscohost.com/login.aspx?direct=true&db=c8h&AN=2010144529&site=ehost-live | Does not meet the review’s definition of Telehealth, which involves the transmission (electronic or verbal) of self-monitored blood glucose (SMBG) readings to a healthcare professional or a specialist team at an offsite monitoring center for evaluation and feedback. |
| 3. Avery L, Flynn D, Van Wersch A, Sniehotta FF, Trenell MI. Changing physical activity behavior in type 2 diabetes: A systematic review and meta-analysis of behavioral interventions. Diabetes Care. 2012;35(12):2681–9. | Does not meet the review’s definition of Telehealth, which involves the transmission (electronic or verbal) of self-monitored blood glucose (SMBG) readings to a healthcare professional or a specialist team at an offsite monitoring center for evaluation and feedback. |
| 4. Azar M, Gabbay R. Web-based management of diabetes through glucose uploads: Has the time come for telemedicine? Diabetes Res Clin Pract. 2009;83(1):9–17. | Not a systematic review |
| 5. Balas EA, Krishna S, Kretschmer RA, Cheek TR, Lobach DF, Boren SA. Computerized knowledge management in diabetes care. Med Care [Internet]. 2004;42(6):610–21. Available from: http://ovidsp.ovid.com/ovidweb.cgi?T=JS&CSC=Y&NEWS=N&PAGE=fulltext&D=med4&AN=15167329%5Cnhttp://lshtmsfx.hosted.exlibrisgroup.com/lshtm?sid=OVID:medline&id=pmid:15167329&id=doi:&issn=0025-7079&isbn=&volume=42&issue=6&spage=610&pages=610-21&date=2004&title=M | T2DM outcomes not reported separately |
| 6. Barlow J, Singh D, Bayer S, Curry R. A systematic review of the benefits of home telecare for frail elderly people and those with long-term conditions. J Telemed Telecare. 2007;13(4):172–9. | T2DM outcomes not reported separately |
| 7. Baron J, McBain H, Newman S. The impact of mobile monitoring technologies on glycosylated hemoglobin in diabetes: a systematic review. J Diabetes Sci Technol [Internet]. 2012;6(5):1185–96. Available from: http://www.ncbi.nlm.nih.gov/pubmed/23063046%5Cnhttp://www.pubmedcentral.nih.gov/articlerender.fcgi?artid=PMC3570854 | Review included trials of mixed study designs (not RCTs only) |
| 8. Biem HJ, Turnell RW, D’Arcy C. Computer telephony: Automated calls for medical care. Clin Investig Med. 2003;26(5):259–68. | T2DM outcomes not reported separately |
| 9. Boren SA, Puchbauer AM, Williams F. Computerized prompting and feedback of diabetes care: a review of the literature. J Diabetes Sci Technol [Internet]. 2009;3(4):944–50. Available from: http://www.pubmedcentral.nih.gov/articlerender.fcgi?artid=2769983&tool=pmcentrez&rendertype=abstract | Unclear what type of diabetes the included study population had. |
| 10. Cassimatis M, Kavanagh DJ. Effects of type 2 diabetes behavioural telehealth interventions on glycaemic control and adherence: a systematic review. J Telemed Telecare [Internet]. 2012;18(8):447–50. Available from: http://www.ncbi.nlm.nih.gov/pubmed/23209266 | Included studies with mixed T1DM and T2DM |
| 11. Chomutare T, Fernandez-Luque L, Arsand E, Hartvigsen G. Features of mobile diabetes applications: Review of the literature and analysis of current applications compared against evidence-based guidelines. J Med Internet Res. 2011;13(3). | T2DM outcomes not reported separately |
| 12. Costa BM, Fitzgerald KJ, Jones KM, Dunning Am T. Effectiveness of IT-based diabetes management interventions: a review of the literature. BMC Fam Pract [Internet]. 2009;10:72. Available from: http://www.pubmedcentral.nih.gov/articlerender.fcgi?artid=2783014&tool=pmcentrez&rendertype=abstract | Review included trials of mixed study designs (not RCTs only) |
| 13. Dale J, Caramlau IO, Lindenmeyer A, Williams SM. Peer support telephone calls for improving health (Review). Cochrane Database Syst Rev. 2009;4(CD006903). | T2DM outcomes not reported separately |
| 14. Dalton JE. *Web-based Care* For Adults with Type 2 Diabetes. Can J Diet Pract Res [Internet]. 2008;69(4):185–91. Available from: http://www.dcjournal.ca/openurl.asp?genre=article&id=doi:10.3148/69.4.2008.185 | Review included trials of mixed study designs (not RCTs only) |
| 15. Dellifraine JL, Dansky KH. Home-based telehealth: a review and meta-analysis. J Telemed Telecare. 2008;14(October 2007):62–6. | T2DM outcomes not reported separately |
| 16. Ekeland, AG., Bowes, A., Flottorp S. Effectiveness of telemedicine: a systematic review of reviews. Int J Med Inform. 2010;79(11):736–71. | T2DM outcomes not reported separately |
| 17. Farmer A, Gibson OJ, Tarassenko L, Neil A. A systematic review of telemedicine interventions to support blood glucose self-monitoring in diabetes. Diabet Med. 2005;22(10):1372–8. | T2DM outcomes not reported separately |
| 18. Gandhi G, Kovalaske M, Kudva Y, Walsh K, Elamin M, Beers M, et al. Efficacy of Continuous Glucose Monitoring in Improving Glycemic Control and Reducing Hypoglycemia: A Systematic Review and Meta-Analysis of Randomized Trials. J Diabetes Sci Technol [Internet]. 2011;5(4):952–65. Available from: http://www.pubmedcentral.nih.gov/articlerender.fcgi?artid=3192603&tool=pmcentrez&rendertype=abstract | Does not meet the review’s definition of Telehealth, which involves the transmission (electronic or verbal) of self-monitored blood glucose (SMBG) readings to a healthcare professional or a specialist team at an offsite monitoring center for evaluation and feedback. |
| 19. Garcia-Lizana F, Sarria-Santamera A. New technologies for chronic disease management and control: a systematic review. J Telemed Telecare [Internet]. 2007;13(2):62–8. Available from: http://jtt.rsmjournals.com/content/13/2/62.full.pdf | T2DM outcomes not reported separately |
| 20. Graziano JA, Gross CR. The effects of isolated telephone interventions on glycemic control in type 2 diabetes. ANS Adv Nurs Sci. 2009;32(3). | Does not meet the review’s definition of Telehealth, which involves the transmission (electronic or verbal) of self-monitored blood glucose (SMBG) readings to a healthcare professional or a specialist team at an offsite monitoring center for evaluation and feedback. |
| 21. Hearnshaw H, Lindenmeyer A, Vermeire E, Van Royen P, Wens J, Biot Y. Interventions to improve adherence to medication in people with type 2 diabetes mellitus: The role of nurses. Eur Diabetes Nurs. 2006;3(2):73–7. | Not an intervention of interest |
| 22. Hersh WR, Helfand M, Wallace J, Kraemer D, Patterson P, Shapiro S, et al. Clinical outcomes resulting from telemedicine interventions: a systematic review. BMC Med Inform Decis Mak [Internet]. 2001;1:5. Available from: http://www.pubmedcentral.nih.gov/articlerender.fcgi?artid=60664&tool=pmcentrez&rendertype=abstract | T2DM outcomes not reported separately |
| 23. Holtz B, Lauckner C. Diabetes Management via Mobile Phones: A Systematic Review. Telemed e-Health. 2012;18(3):175–84. | Review included trials of mixed study designs (not RCTs only) |
| 24. Hunt DL, Haynes RB, Hayward RSA, Pim MA, Horsman J. Patient-specific evidence-based care recommendations for diabetes mellitus: Development and initial clinic experience with a computerized decision support system. Int J Med Inform. 1998;51(2–3):127–35. | T2DM outcomes not reported separately |
| 25. Jaana M, Paré G. Home telemonitoring of patients with diabetes: A systematic assessment of observed effects. J Eval Clin Pract. 2007;13(2):242–53. | T2DM outcomes not reported separately |
| 26. Jackson CL, Bolen S, Brancati FL, Batts-Turner ML, Gary TL. A systematic review of interactive computer-assisted technology in diabetes care: Interactive information technology in diabetes care. J Gen Intern Med. 2006;21(2):105–10. | T2DM outcomes not reported separately |
| 27. Jalil S, Myers T, Atkinson I. A Meta-Synthesis of Behavioral Outcomes from Telemedicine Clinical Trials for Type 2 Diabetes and the Clinical User-Experience Evaluation (CUE). J Med Syst. 2015;39(3). | Review included trials of mixed study designs (not RCTs only) |
| 28. Jongh D. Cochrane Database of Systematic Reviews Mobile phone messaging for facilitating self-management of long-term illnesses ( Review ). Cochrane Libr. 2012;(12). | Unclear what type of diabetes the included study population had. |
| 29. Kaplan RM, Stone AA. Bringing the Laboratory and Clinic to the Community: Mobile Technologies for Health Promotion and Disease Prevention a. Annu Rev Psychol [Internet]. 2013;64:471–98. Available from: www.annualreviews.org | Not a systematic review |
| 30. Liang X, Wang Q, Yang X, Cao J, Chen J, Mo X, et al. Effect of mobile phone intervention for diabetes on glycaemic control: A meta-analysis. Diabet Med. 2011;28(4):455–63. | Review included trials of mixed study designs (not RCTs only) |
| 31. Marcolino MS, Maia JX, Alkmim MBM, Boersma E, Ribeiro AL. Telemedicine application in the care of diabetes patients: Systematic review and meta-analysis. PLoS One. 2013;8(11):1–13. | T2DM outcomes not reported separately |
| 32. Medical Advisory Secretariat. Home telemonitoring for type 2 diabetes: an evidence-based analysis. Ont Health Technol Assess Ser [Internet]. 2009;9(24):1–38. Available from: http://www.pubmedcentral.nih.gov/articlerender.fcgi?artid=3377533&tool=pmcentrez&rendertype=abstract%5Cnhttp://www.ncbi.nlm.nih.gov/pubmed/23074529%5Cnhttp://www.pubmedcentral.nih.gov/articlerender.fcgi?artid=PMC3377533 | T2DM outcomes not reported separately |
| 33. Mignerat M, Lapointe L, Vedel I. Using telecare for diabetic patients: A mixed systematic review. Heal Policy Technol [Internet]. 2014;3(2):90–112. Available from: http://dx.doi.org/10.1016/j.hlpt.2014.01.004 | Review included trials of mixed study designs (not RCTs only) |
| 34. Norris SL, Lau J, Smith SJ, Schmid CH, Engelgau MM. Self-Management Education for Adults With Type 2 Diabetes: A meta-analysis of the effect on glycemic control. Diabetes Care [Internet]. 2002;25(7):1159–71. Available from: http://care.diabetesjournals.org/content/25/7/1159.short | Does not meet the review’s definition of Telehealth, which involves the transmission (electronic or verbal) of self-monitored blood glucose (SMBG) readings to a healthcare professional or a specialist team at an offsite monitoring center for evaluation and feedback. |
| 35. Pal K, Eastwood S, Michie S. Computer‐based diabetes self‐management interventions for adults with type 2 diabetes mellitus. Cochrane Libr. 2010;(3). | Does not meet the review’s definition of Telehealth, which involves the transmission (electronic or verbal) of self-monitored blood glucose (SMBG) readings to a healthcare professional or a specialist team at an offsite monitoring center for evaluation and feedback. |
| 36. Paré G, Jaana M, Sicotte C, Paper R. Systematic review of home telemonitoring for chronic diseases: the evidence base. J Am Med Inform Assoc [Internet]. 2007;14(3):269–77. Available from: http://dx.doi.org/10.1197/jamia.m2270 | T2DM outcomes not reported separately |
| 37. Polisena J, Tran K, Cimon K, Hutton B, McGill S, Palmer K. Home telehealth for diabetes management: A systematic review and meta-analysis. Diabetes, Obes Metab. 2009;11(10):913–30. | T2DM outcomes not reported separately |
| 38. Ramadas A, Quek KF, Chan CKY, Oldenburg B. Web-based interventions for the management of type 2 diabetes mellitus: A systematic review of recent evidence. Int J Med Inform [Internet]. 2011;80(6):389–405. Available from: http://dx.doi.org/10.1016/j.ijmedinf.2011.02.002 | Does not meet the review’s definition of Telehealth |
| 39. Sarol JN, Nicodemus NA, Tan KM, Grava MB. Self-monitoring of blood glucose as part of a multi-component therapy among non-insulin requiring type 2 diabetes patients: a meta-analysis (1966-2004). Curr Med Res Opin [Internet]. 2005;21(2):173–84. Available from: http://www.ncbi.nlm.nih.gov/pubmed/15801988 | Does not meet the review’s definition of Telehealth, which involves the transmission (electronic or verbal) of self-monitored blood glucose (SMBG) readings to a healthcare professional or a specialist team at an offsite monitoring center for evaluation and feedback. |
| 40. Singh D, Ham C. Transforming chronic care. Evidence about improving care for people with long-term conditions [Internet]. 2005. Available from: http://www.download.bham.ac.uk/hsmc/pdf/transforming_chronic_care.pdf | T2DM outcomes not reported separately |
| 41. Siriwardena LS a. N, Wickramasinghe W a. S, Perera KLD, Marasinghe RB, Katulanda P, Hewapathirana R. A review of telemedicine interventions in diabetes care. J Telemed Telecare. 2012;18(3):164–8. | T2DM outcomes not reported separately |
| 42. Soe K, Sacerdote A, Karam J, Bahtiyar G. Management of type 2 diabetes mellitus in the elderly. Maturitas [Internet]. 2011;70(2):151–9. Available from: http://dx.doi.org/10.1016/j.maturitas.2011.07.006 | Does not meet the review’s definition of Telehealth, which involves the transmission (electronic or verbal) of self-monitored blood glucose (SMBG) readings to a healthcare professional or a specialist team at an offsite monitoring center for evaluation and feedback. |
| 43. Sutcliffe P, Martin S, Sturt J, Powell J, Griffiths F, Adams A, et al. Systematic review of communication technologies to promote access and engagement of young people with diabetes into healthcare. BMC Endocr Disord [Internet]. 2011;11(1):1. Available from: http://www.pubmedcentral.nih.gov/articlerender.fcgi?artid=3024230&tool=pmcentrez&rendertype=abstract | T2DM outcomes not reported separately |
| 44. Swinnen SGHA, Devries JH. Contact frequency determines outcome of basal insulin initiation trials in type 2 diabetes. Diabetologia. 2009;52(11):2324–7. | Not a systematic review and does not meet the review’s definition of Telehealth, which involves the transmission (electronic or verbal) of self-monitored blood glucose (SMBG) readings to a healthcare professional or a specialist team at an offsite monitoring center for evaluation and feedback. |
| 45. Toma T, Athanasiou T, Harling L, Darzi A, Ashrafian H. Online social networking services in the management of patients with diabetes mellitus: Systematic review and meta-analysis of randomised controlled trials. Diabetes Res Clin Pract [Internet]. 2014;106(2):200–11. Available from: http://dx.doi.org/10.1016/j.diabres.2014.06.008 | Does not meet the review’s definition of Telehealth, which involves the transmission (electronic or verbal) of self-monitored blood glucose (SMBG) readings to a healthcare professional or a specialist team at an offsite monitoring center for evaluation and feedback. |
| 46. Tran K, Polisena J, Coyle D, Coyle K, Kluge EH, Cimon K, et al. Home telehealth for chronic disease management [Internet]. Ottawa: Canadian Agency for Drugs and Technologies in Health; 2008. 1 p. Available from: http://onlinelibrary.wiley.com/o/cochrane/cldare/articles/DARE-12010008017/frame.html | T2DM outcomes not reported separately |
| 47. Upadhyay N, Kokalj Kokot M, Kokalj Kokot M, Svab I, Car J. Mobile phone messaging - A telemedicine for people with diabetes mellitus. Cochrane Database Syst Rev. 2007;(1). | T2DM outcomes not reported separately |
| 48. Van Den Berg N, Schumann M, Kraft K, Hoffmann W. Telemedicine and telecare for older patients—A systematic review. Maturitas. 2012;73:94–114. | T2DM outcomes not reported separately |
| 49. Verhoeven F, Tanja-Dijkstra K, Nijland N, Eysenbach G, Van Gemert-Pijnen L. Asynchronous and synchronous teleconsultation for diabetes care: A systematic literature review. J Diabetes Sci Technol [Internet]. 2010;4(3):666–84. Available from: https://www.scopus.com/inward/record.uri?eid=2-s2.0-77958486329&partnerID=40&md5=380a8490d770c59aa0459ade9adb0c2c | T2DM outcomes not reported separately |
| 50. Verhoeven F, Van Gemert-Pijnen L, Dijkstra K, Nijland N, Seydel E, Steehouder M. The contribution of teleconsultation and videoconferencing to diabetes care: A systematic literature review. J Med Internet Res. 2007;9(5). | T2DM outcomes not reported separately |
| 51. Vodopivec-Jamsek V, De Jongh T, Gurol-Urganci I, Atun R, Car J. Mobile phone messaging for preventive health care (Intervention Review). Cochrane Libr. 2012;(12). | T2DM outcomes not reported separately |
| 52. Wu L, Forbes A, Griffiths PD, While A. Telephone follow-up for type 2 diabetes mellitus. Cochrane Database Syst Rev. 2007;(3). | Unclear if the included trials meet the review’s definition of Telehealth, which involves the transmission (electronic or verbal) of self-monitored blood glucose (SMBG) readings to a healthcare professional or a specialist team at an offsite monitoring center for evaluation and feedback. |
| 53. Yu CH, Bahniwal R, Laupacis A, Leung E, Orr MS, Straus SE. Systematic review and evaluation of web-accessible tools for management of diabetes and related cardiovascular risk factors by patients and healthcare providers. J Am Med Inf Assoc. 2012;19(4):514–22. | T2DM outcomes not reported separately |
| 54. Zhai Y, Zhu W, Cai Y, Sun D, Zhao J. Clinical- and cost-effectiveness of telemedicine in type 2 diabetes mellitus: a systematic review and meta-analysis. Medicine (Baltimore) [Internet]. 2014;93(28):e312. Available from: http://www.scopus.com/inward/record.url?eid=2-s2.0-84920124758&partnerID=tZOtx3y1 | Does not meet the review’s definition of Telehealth, which involves the transmission (electronic or verbal) of self-monitored blood glucose (SMBG) readings to a healthcare professional or a specialist team at an offsite monitoring center for evaluation and feedback. |
