# Supplementary material for: The impact of telehealth remote patient monitoring on glycemic control in type 2 diabetes: a systematic review and meta-analysis of systematic reviews of randomised controlled trials
Source: BMC Health Serv Res. 2018 Jun 26;18:495. doi: 10.1186/s12913-018-3274-8 (PMC6019730; doi:10.1186/s12913-018-3274-8)
Supplement: Supplementary file 4 — Evidence tables. (DOCX 98 kb) [file 12913_2018_3274_MOESM4_ESM.docx]

**Additional file 4. Evidence Tables**

| **Reference** | **Characteristics of review** | **Types of telehealth interventions** | **Types of telehealth applications/ technologies** | **Types of feedback methods** | **Summary of results** |
| --- | --- | --- | --- | --- | --- |
| **Greenwood (2014)**  **Title:** Telehealth remote monitoring systematic review: Structured self-monitoring of blood glucose and impact on A1C.  **Aim of review:** *“To summarize research on telehealth remote patient monitoring interventions that incorporate key elements of structured self-monitoring of blood glucose (SMBG) identified as essential for improving A1C”.* | Included studies were published between (year): 2005 and 2013  No. of participants (n): 4468  No. of studies (n): 15  Follow-up time: Ranged from 3 to 60 months  Countries in which the included studies were conducted in: Not reported | Telemonitoring: n=8  Telemonitoring + e-learning: n=1  Telemonitoring + e-learning + social networking support group: n=1  Telemonitoring + virtual coach: n=1  Telemonitoring + Online/phone secure messaging with team + e-learning: n=2  Telemonitoring + secure messaging with team + e-learning + videoconferencing: n=1  Telemonitoring + virtual coach + secure messaging with team + e-learning: n=1 | Mobile phone: n=4  Telephone: =1  Computer or Internet-based platform: n=8  Computer/web + phone/mobile: n=2 | Telephone calls: n=1  Videoconferencing: n=2  Mobile phones using short message service (SMS) or text messaging: n=3  Secure messages via a patient portal or telehealth system: n=2  Automated feedback generated from computer algorithms, without provider input: n=2  Phone, letter or home telemonitoring unit: n=1  Not reported/ Unclear: n=4 | **HbA1c** 10 of the 15 RCTs reported statistically significant improvements in HbA1c between groups.  **Authors' conclusions:** “*Telehealth remote patient monitoring interventions in type 2 diabetes have not included all structured monitoring elements recommended by the International Diabetes Federation. Incorporating more elements of structured SMBG is associated with improved A1C”.* |
| **Huang (2015)**  **Title:** Management of endocrine disease. Effects of telecare intervention on glycemic control in type 2 diabetes: a systematic review and meta-analysis of randomized controlled trials.  **Aim of review:** *“The aim of the review To review the published literature on the effects of telecare intervention in patients with type 2 diabetes and inadequate glycemic control”.* | Included studies were published between (year): 2000 and 2013  No. of participants (n): 3798  No. of studies (n): 18  Follow-up time: 3 to 60 months  Countries in which the included studies were conducted in: Not reported | Internet-based platform: n=11  Telephone/ Mobile: n=7 | Internet-based platform: n=11  Telephone/ Mobile: n=7 | Telephone calls: n=5  Automated calls of prerecorded voice message from call centre: n=2  Automated internet text messages and SMS: n=9 | **HbA1c:** An analysis of 18 studies showed that telecare significantly improved HbA1c levels when compared to standard care (-0.54, 95% CI: -0.75 to -0.34), with human calls subgroup being associated with the greatest effect size  ***Authors' conclusions:***  *“Patients monitored by telecare showed significant improvement in glycemic control in type 2 diabetes when compared with those monitored by routine follow-up. Significant reduction in HbA1c levels was associated with Asian populations, small sample size, and telecare, and with those patients with baseline HbA1c greater than 8%”.* |
| **Russell-Minda (2009)**  **Title:** Health technologies for monitoring and managing diabetes: a systematic review  **Aim of the review:** *“To determine the strength of evidence for the effectiveness of self-monitoring devices and technologies for individuals with type 1 diabetes mellitus (T1DM) or type 2 diabetes mellitus (T2DM) based on specific health-related outcome measures”.* | Included studies were published between (year): 2007 and 2008  No. of participants (n): 894  No. of studies (n): 5 T2DM  Follow-up time: 3 to 12 months  Countries in which the included studies were conducted in: Not reported | Telemonitoring n=5 (some with nurse-directed educational component) | Mobile phone:  n=1  Computer/web + phone/mobile: n=4 | Mobile phones using short message service (SMS) or text messaging: n=4  Not reported/ Unclear: n=1 | **HbA1c** 3 (2 moderately strong and 1 good quality) out of 5 RCTs reported significant improvements in HbA1c in the TH intervention group when compared to standard care group.  ***Authors' conclusions:***  “*Wireless technologies may improve diabetes self-care. The results of this review indicate a need for additional controlled trial research on existing and novel technologies for diabetes self-monitoring, on health outcomes associated with diabetes and CVCs, and device feasibility and compliance”.* |
| **Tildesley (2015)**  **Title:** Internet blood glucose monitoring systems provide lasting glycemic benefit in type 1 and 2 diabetes: a systematic review.  **Aim of the review:** “*To evaluate the efficacy, safety and other outcomes from numerous small studies comparing patients using internet blood glucose monitoring systems (IBGMS), which facilitate regular health care provider review and feedback regarding a patient's SMBG results, with other patients with more traditional patterns of physician contact”.* | Included studies were published between (year): 2004 and 2011  No. of participants (n): 1371  No. of studies (n): 9  Follow-up time:  3 to 60 months  Countries in which the included studies were conducted in: Not reported | Internet-based blood glucose monitoring systems: All studies | Home-based TH unit: n=1  Computer or Internet-based platform: n=6  Wireless glucometer: n=2 | Telephone calls: n=2  Mobile phones using short message service (SMS) or text messaging: n=2  Internet/web-based: n=4 Internet/web-based and phone/mobile: n=1  Email: n=1 | **HbA1c** 7 out of 9 RCTs reported significantly improved HbA1c level in the IBGMS group compared with the usual care group. 2 RCTs reported significant improvements in HbA1c levels in the intervention group. A mean baseline HbA1c level 8% or greater was associated with the most remarkable improvements in glycemic control with TH intervention.  ***Authors' conclusions:*** *“Available study data on IBGMS support the clinical usefulness of this approach to diabetes management, with improved glycemic control in all types of diabetes patients and no increase in hypoglycemia risk.*  *Glycemic improvements likely result from a combination of factors, including increased patient motivation and increased communication between patient and health care provider”.* |
