# Supplementary material for: The impact of telehealth remote patient monitoring on glycemic control in type 2 diabetes: a systematic review and meta-analysis of systematic reviews of randomised controlled trials
Source: BMC Health Serv Res. 2018 Jun 26;18:495. doi: 10.1186/s12913-018-3274-8 (PMC6019730; doi:10.1186/s12913-018-3274-8)
Supplement: Supplementary file 5 — NMAs on HbA1c by telehealth transmission methods. (DOCX 89 kb) [file 12913_2018_3274_MOESM5_ESM.docx]

**Additional file 5. Network meta-analyses on HbA1c by telehealth application/transmission methods**

|  | **Automatic transmission (=5)** | **Automatic mobile transmission (n=6)** | **Internet/web (n=9)** | **Telephone (human/automatic (n=5)** | **Usual care** |
| --- | --- | --- | --- | --- | --- |
| **Automatic transmission (via modem or Bluetooth) (n=5)** |  | -0.0920  [-0.5625 to 0.3785] | 0.4015  [-0.0578 to 0.8607] | 0.3514  [-0.1876 to 0.8904] | -0.3783  [-0.7291 to -0.0276]* |
| **Automatic mobile transmission (via mobile phones) (n=6)** | 0.0920  [-0.3785 to 0.5625] |  | 0.4934  [0.0619 to 0.9250] | 0.4434  [-0.0722 to 0.9590] | -0.8264  [-0.6000 to 0.0273] |
| **Internet/web (via mobile phone or computer) (n=9)** | -0.4015  [-0.8607 to 0.0578] | -0.4934  [-0.9250 to -0.0619]* |  | -0.05000  [-0.5554 to 0.4553] | -0.7798  [-1.0762 to -0.4833]* |
| **Telephone (automated/human) (n=5)** | -0.3514  [-0.8904 to 0.1876] | -0.4434  [-0.9590 to 0.0722] | 0.0500  [-0.4553 to 0.5554] |  | -0.7298  [-1.1390 to -0.3205]* |
| **Usual care** | 0.3783  [0.0276 to 0.7291] | 0.2864  [-0.0273 to 0.6000] | 0.7798  [0.4833 to 1.0762] | 0.7298  [0.3205 to 1.1390] |  |

I^2^ = 75.3%

*Significant difference between groups
