# Supplementary material for: The impact of telehealth remote patient monitoring on glycemic control in type 2 diabetes: a systematic review and meta-analysis of systematic reviews of randomised controlled trials
Source: BMC Health Serv Res. 2018 Jun 26;18:495. doi: 10.1186/s12913-018-3274-8 (PMC6019730; doi:10.1186/s12913-018-3274-8)
Supplement: Supplementary file 6 — NMAs on HbA1c by telehealth feedback methods. (DOCX 119 kb) [file 12913_2018_3274_MOESM6_ESM.docx]

**Additional file 6. Network meta-analyses on HbA1c by telehealth feedback methods**

|  | **Automated message (SMS/online) (n=6)** | **Human calls (n=5)** | **Human calls only if necessary (n=3)** | **Human message (SMS/online) (n=9)** | **Human message (SMS/online) + calls (n=1)** | **Videoconferencing (n=1)** | **Usual care** |
| --- | --- | --- | --- | --- | --- | --- | --- |
| **Automated message (SMS/online)** |  | 0.4838  [-0.1498 to 1.1175 | -0.4930  [-1.1957 to 0.2098 | 0.2101  [-0.3208 to 0.7411 | -0.1745  [-1.2741 to 0.9525 | -0.1845  [-1.1875 to 0 .8186] | -0.4745  [-0.8815 to -0.0674]* |
| **Human calls** | -0.4838  [-1.1175 to 0.1498] |  | -0.9768  [-1.7278 to -0.2285]* | -0.2737  [-0.8670 to 0.3196] | -0.6583  [-1.7894 to 0.4728] | -0.6683  [-1.7057 to 0.3691] | -0.9583  [-1.4439 to -0.4727]* |
| **Human calls only if necessary** | 0.4930  [-0.2098 to 1.1958] | 0.9768  [0.2258 to 1.7278] |  | 0.7031  [0.0365 to 1.3697] | 0.3185  [-0.8527 to 1.4898] | 0.3085  [-0.7725 to 1.3895] | 0.0185  [-0.5544 to 0.5914] |
| **Human message (SMS/online)** | -0.2101  [-0.7411 to 0.3208] | 0.2737  [-0.3196 to 0.8670] | -0.7031  [-1.3697 to -0.0365]* |  | -0.3846  [-1.4615 to 0.6923] | -0.3946  [-1.3727 to 0.5835] | -0.6846  [-1.0255 to -0.3437]* |
| **Human message (SMS/online) + calls** | 0.1745  [-0.8252 to 1.2741] | 0.6583  [-0.4728 to 1.7894] | -0.3185  [-1.4898 to 0.8527] | 0.3846  [-0.6923 to 1.4615] |  | -0.0100  -1.3826 to 1.3626] | -0.3000  [-1.3216 to 0.7216] |
| **Videoconferencing** | 0.1845  [-0.8486 to 1.1875] | 0.6683  [-0.3691 to 1.7057] | -0.3085  [-1.3895 to 0.7725] | 0.3946  [-0.5835 to 1.3727] | 0.0100  [-1.3626 to 1.3826] |  | -0.2900  [-1.2067 to 0.6267] |
| **Usual care** | 0.4745  [0.0674 to 0.8815] | 0.9583  [0.4727 to 1.4439] | -0.0185  [-0.5914 to 0.5544] | 0.6846  [0.3437 to 1.0255] | 0.3000  [-0.7216 to 1.3216 | 0.2900  [-0.6267 to 1.2067 |  |

I^2^ = 82.3%

*Significant difference between groups
